# Seroepidemiological Study of Toxocariasis in Children Aged 6–14 Year Old in Sanandaj, Western Iran

**DOI:** 10.18502/ijpa.v15i3.4209

**Published:** 2020

**Authors:** Yahya MAROUFI, Ashkan FARIDI, Mohammadbagher KHADEMERFAN, Fares BAHRAMI, Ghasem ZAMINI

**Affiliations:** 1. Department of Parasitology and Mycology, Faculty of Medicine, Kurdistan University of Medical Sciences, Sanandaj, Iran; 2. Zoonoses Research Center, Research Institute for Health Development, Kurdistan University of Medical Sciences, Sanandaj, Iran

**Keywords:** Children, *Toxocara*, Toxocariasis, Iran

## Abstract

**Background:**

Toxocariasis is a disease caused by *Toxocara* nematodes and occurs from consuming their eggs. The main hosts of these worms are dogs and cats. The disease in humans becomes a visceral larva migrans (VLM). This descriptive cross-sectional study was conducted to determine the prevalence of toxocariasis in children aged 6–14 years.

**Methods:**

This cross-sectional descriptive study was conducted from Jun 1 2016 to Dec 1 2017 in Sanandaj, west of Iran. A total of 182 serum samples were collected from children age 6 to14 yr referred to medical diagnostic laboratories. Demographic data (age, sex, and parents’ literacy status), clinical signs (cough, headache, fever, abdominal pain), and the history of contact with dogs and cats was collected by a questionnaire. The presence of anti-*Toxocara* IgG antibody was detected by *T. canis* IgG ELISA (IBL, Germany) kit.

**Results:**

Of 182 subjects, 97 (53.3%) were male and 85 (46.7%) female. The average age was 9.2 years. Antibodies against *T. canis* were positive in three cases (1.65%) of all the studied subjects.

**Conclusions:**

The results showed a low prevalence of toxocariasis in children studied.

## Introduction

Toxocariasis is caused by a series of nematode species (called ascarids or *Toxocara* spp.) that routinely infect dogs and cats. *T*. *canis* (dog roundworm) is the main cause of the disease in humans. Human toxocariasis is most prevalent helminthozoonosis diseases caused by *T. canis* and *T. cati*, due to the migration larvae through human organism.

The *Toxocara* eggs pass out of the body of the definitive host in the excreta feces, the embryonation of eggs occurs in the soil (1). Stray dogs are considered as the most important causes of outbreaks in the environment; in the process of they disperse feces in the city’s parks, on the streets, or playgrounds, and in private gardens (2). Humans become an accidental host because of ingesting the eggs containing the larval stage of the *Toxocara*. Young children (aged 6–14 yr) have a higher chance of being exposed to the parasite eggs due to play and behavioral habits, usually by touching contaminated hands to the mouth or direct and close contact with the soil and dogs (3–5). Humans are infected by ingesting the eggs from contaminated soil, food, or water; and by eating undercooked meat of paratenic hosts like chicken, cow, and sheep (6, 7). The eggs hatch in the duodenum and the larvae penetrate the intestinal wall, then they are carried by the circulation system to a wide variety of tissues (liver, heart, lungs, brain, muscle, and eyes). *Toxocara* larvae tend to accumulate in an organ, especially in the CNS, causing a chronic disease (2).

The signs and symptoms vary with regard to the number of migrating juveniles and the involved organs. There are three clinical manifestation of *Toxocara* infection in humans as follows: 1) visceral toxocariasis or visceral larva migrans (VLM); 2) ocular toxocariasis or ocular larva migrans (OLM); 3) nervous toxocariasis or nervous larva migrans (NLM); and 4) covert or common toxocariasis (CT), is usually manifested as mild and non-specific. VLM symptoms usually include fever, cough, wheezing, hepatomegaly, splenomegaly, fatigue, malnutrition, anorexia, and abdominal pain. Toxocariasis is related to asthma, pulmonary inflammation, and eosinophilia in children. In addition, some of the complications of toxocariasis in the central nervous system have been reported as convulsions and mental and growth retardation (3, 8, 9). The migration of *Toxocara* larva to the eye (OLM) causes reduced visual acuity, strabismus, and, in some cases, blindness in one or both eyes due to granulomatous lesions in the retina. OLM in most cases occur in children aged 5– 10 yr (10). Serological techniques are reliable methods to detect larval antigens. Until now ELISA with *Toxocara* excretory–secretory antigens (TES-ELISA) is the most reliable methods to detect *Toxocara*-specific antibodies (11).

The prevalence of toxocariasis was reported in different countries as follows: Bolivia 27%, Brazil 8.7–38.8%, Chile 2.2%, Colombia 47.5%, Cuba 5.2%, Puerto Rico 6.5%, Venezuela 66.6%, Peru 32.4%, Ecuador 30%, Argentina 10.6–36.9%, Korea 50.5%, Spain 1%, and Denmark 2.4% (12–15). Based on previous data, the average seroprevalence of *Toxocara* infection from 2000 to 2010 among Iranian children was 15.8% (16).

Various factors, such as the presence of stray dogs in the city and the easy entry of dogs into parks may bring the spread of *Toxocara* eggs. One of the main problems for controlling and eradicating toxocariasis is the lack of accurate statistics for its prevalence. Therefore, this study was conducted to determine the prevalence of toxocariasis in children aged 6–14 yr in Sanandaj, Iran in 2017.

## Materials and Methods

**Fig. 1:**
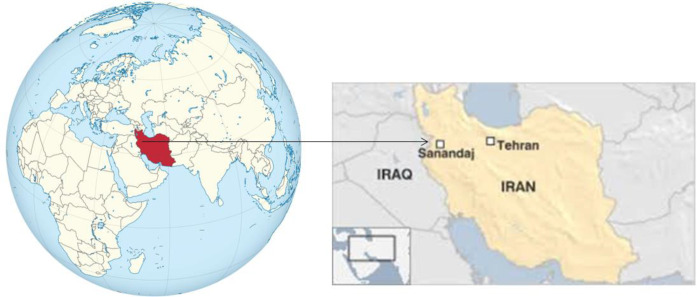
Geographical location of Sanandaj city in west of Iran

This cross-sectional descriptive study was conducted from Jun 1 2016 to Dec 1 2017 in Sanandaj. Sanandaj is the capital town of Kurdistan province in Iran with geographical coordinates of 35°20′ north, 47°18′ east of Greenwich meridian, and 15° west of Tehran meridian (Fig. 1) (17).

During Jun to Dec 2017, 182 serum samples were collected from children aged between 6 and 14 yr who referred to medical diagnostic laboratories. Samples were kept at −20 °C until the test was performed. Demographic data and history of contact with dogs were obtained by questionnaire.

The presence of anti-*Toxocara* IgG antibody of *T. canis* was detected by using IgG ELISA kit (IBL, Germany) and performed according to the manufacturer’s instructions.

STATA version 12.0 (Stata Corp, College Station, Texas USA) was used to analyze the data of the study. At first, the prevalence of contamination was assessed in general and then in terms of the variables considered. For all estimated values, a 95% confidence interval was also calculated.

### Ethical approval

The present study was performed in accordance with the ethical standards of the Declaration of Helsinki. It was approved by the Ethics Committee of the Kurdistan University of Medical Sciences (Grant No. 1395/190).

## Results

Of 182 samples, 97 (53.3%) were male and 85 (46.7%) female. The mean and standard deviation of the participants in the study were 9.2 and 2.6 yr, respectively. Three cases (95% CI = 0.349: 95% CI, *P* = 0.0165) showed antibodies against *T. canis*. Of them, two were female (95% CI-057/05%, *P* = 0.0235), and one was male (95% CI-0317 / 0– 95%, *P* = 0.0103). The mean and standard deviation of the age of the positive cases were 8 and 1 yr, respectively. Of these, 2 showed a history of contact with dogs. Abdominal pain was present in all three positive cases. Cough and headache was reported in the 2 positive cases.

## Discussion

The average seroprevalence of toxocariasis in Iranian children is 15.8%. In this study, the prevalence rate was 1.64%. The seroprevalence of toxocariasis is similar to the results in urban area in Zanjan (1.6%) (18), Isfahan (1.39%) (19) and Ahwaz (2%) (16, 20). However, higher prevalences were reported in Sistan and Baluchestan (3.8%), Shiraz (25.6%) (21), East Azerbaijan (29.46%) (22), Hamedan (8.8%) (23), Sari (25%) (24), Lorestan (4.4%) (25) and Mahidasht (46.8%) (26).

Dogs are definitive hosts of *T. canis* and are a major factor in the spread of eggs in the environment. In another study conducted by the authors, the contamination rate in stray dogs in Sanandaj was 6.3% in 2017 (unpublished data). Otherwise, the average infection rate in dogs in Iran was 26.8% (16, 20). Therefore, a low prevalence in children in this study is related to the rate of infection in stray dogs within the geographical region of Sanandaj.

In this study, out of 3 positive cases, 2 cases were girls and 2 cases had a history of contact with dogs. There was no significant relationship between age, sex, history of contact with dogs, and the level of education of the parents. Abdominal pain was reported in all positive cases, cough and headache were reported in 2 out of the 3 positive cases. Fever was not seen in any of the positive cases.

Cultural differences such as the absence shepherds and domestic dogs can be attributed to the low prevalence in children who live in Sanandaj when compared with other areas.

## Conclusion

This study shows a low prevalence of toxocariasis in children in Sanandaj at 2017.

## Ethical considerations

Ethical issues (Including plagiarism, informed consent, misconduct, data fabrication and/or falsification, double publication and/or submission, redundancy, etc.) have been completely observed by the authors.
